# Dichloridobis[2-(1-hydrazinylideneeth­yl)pyrazine-κ*N* ^1^]zinc

**DOI:** 10.1107/S1600536812016418

**Published:** 2012-04-28

**Authors:** Li-li Liang, Meng Li, Jia-cheng Liu, Yun Wei

**Affiliations:** a College of Chemistry and Chemical Engineering, Northwest Normal University, Lanzhou 730070, People’s Republic of China

## Abstract

In the structure of the title complex, [ZnCl_2_(C_6_H_8_N_4_)_2_], the Zn^II^ atom has a distorted octa­hedral geometry. Two *cis* Cl^−^ ions and four N atoms belonging to two different 2-(1-hydrazinylideneeth­yl)pyrazine ligands coordinate the Zn^II^ atom, forming two five-membered Zn—N—C—C—N rings. The dihedral angle between the planes of these metallocycles is 88.13 (4)°. The organic ligands are essentially planar (r.m.s. deviations from planarity = 0.072 and 0.040 Å). Inter­molecular N—H⋯N and N—H⋯Cl inter­actions join the mol­ecules into a three-dimensional framework.

## Related literature

For the biochemical applications of complexes based on ligands containing pyrazine, see: Ha *et al.* (1999 ▶); Blackstock *et al.* (2000 ▶); Adams *et al.* (2002 ▶); Lee *et al.* (2012 ▶). For the preparation of the ligand, see: Stadler *et al.* (2010 ▶).
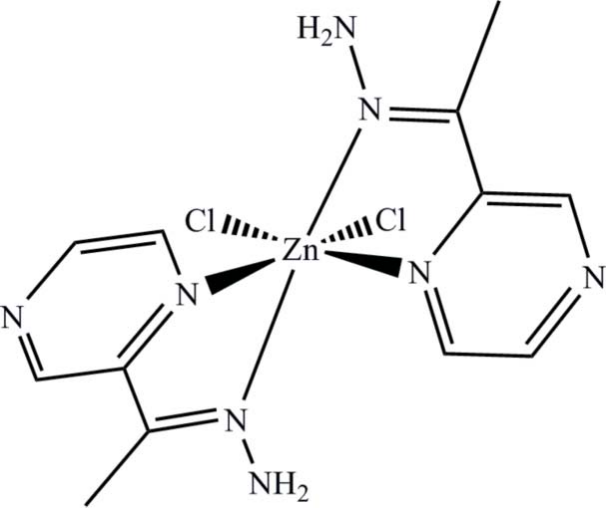


## Experimental

### 

#### Crystal data

[ZnCl_2_(C_6_H_8_N_4_)_2_]
*M* *_r_* = 408.60
Monoclinic, 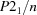

*a* = 8.4289 (8) Å
*b* = 15.1128 (14) Å
*c* = 13.4196 (13) Å
β = 104.077 (1)°
*V* = 1658.1 (3) Å^3^
*Z* = 4
Mo *K*α radiation
μ = 1.81 mm^−1^
*T* = 296 K
0.20 × 0.18 × 0.15 mm

#### Data collection

Bruker APEXII CCD diffractometer
Absorption correction: multi-scan (*SADABS*; Bruker, 2008 ▶) *T*_min_ = 0.713, *T*_max_ = 0.773
11576 measured reflections
4132 independent reflections
3456 reflections with *I* > 2σ(*I*)
*R*_int_ = 0.026

#### Refinement

*R*[*F* ^2^ > 2σ(*F* ^2^)] = 0.029
*wR*(*F* ^2^) = 0.078
*S* = 1.03
4132 reflections
223 parameters
4 restraints
H atoms treated by a mixture of independent and constrained refinement
Δρ_max_ = 0.42 e Å^−3^
Δρ_min_ = −0.21 e Å^−3^

### 

Data collection: *APEX2* (Bruker, 2008 ▶); cell refinement: *SAINT-Plus* (Bruker, 2008 ▶); data reduction: *SAINT-Plus*; program(s) used to solve structure: *SHELXS97* (Sheldrick, 2008 ▶); program(s) used to refine structure: *SHELXL97* (Sheldrick, 2008 ▶); molecular graphics: *XP* in *SHELXTL* (Sheldrick, 2008 ▶); software used to prepare material for publication: *SHELXTL*.

## Supplementary Material

Crystal structure: contains datablock(s) I, global. DOI: 10.1107/S1600536812016418/pk2404sup1.cif

Structure factors: contains datablock(s) I. DOI: 10.1107/S1600536812016418/pk2404Isup2.hkl

Additional supplementary materials:  crystallographic information; 3D view; checkCIF report

## Figures and Tables

**Table 1:** Hydrogen-bond geometry (Å, °)

*D*—H⋯*A*	*D*—H	H⋯*A*	*D*⋯*A*	*D*—H⋯*A*
N8—H2*N*8⋯Cl1	0.86 (2)	2.73 (2)	3.400 (2)	137 (2)
N8—H2*N*8⋯N1^i^	0.86 (2)	2.60 (2)	3.165 (3)	124 (2)
N4—H2*N*4⋯Cl2	0.83 (2)	2.60 (2)	3.250 (2)	137 (2)
N4—H1*N*4⋯Cl1^ii^	0.81 (2)	2.66 (2)	3.4429 (19)	165 (2)

Symmetry codes: (i) 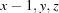; (ii) 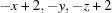.

**Fig. 1.**
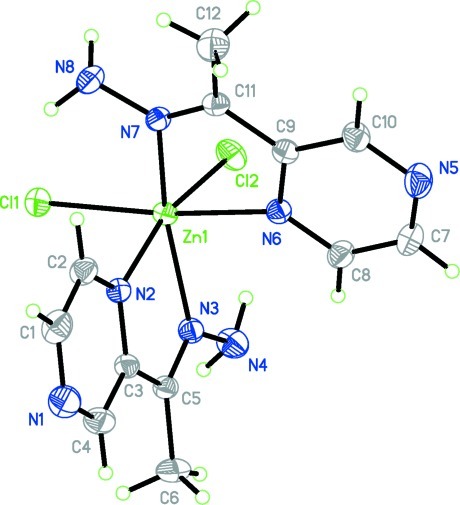
The crystal structure of the title complex. Ellipsoids are drawn at the 50% probability level. Hydrogen atoms are depicted as small spheres of arbitrary radius.

**Fig. 2.**
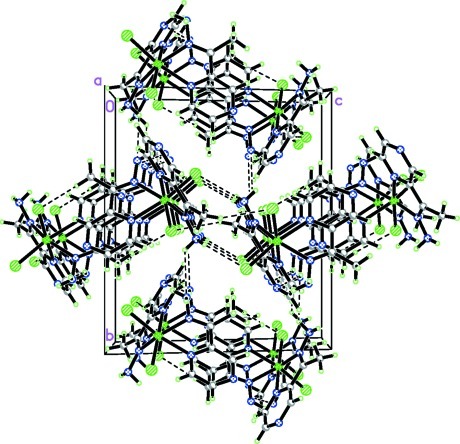
The packing diagram of the title complex, viewed along the a axis. Intermolecular interactions are shown as dashed lines.

## supplementary crystallographic information

### Comment

Pyrazine and its derivatives have received considerable attention and interest in the field of biochemistry. Such research has been focused on drugs (Ha *et al.*, 1999; Blackstock *et al.*, 2000), flavor ingredients (Adams *et al.*, 2002), and enzymatic modification (Lee *et al.*, 2012). In this paper, a new Zn^II^ complex, dicholorido-bis[2-(1-hydrazonethyl) pyrazine]zinc(II), is described.

The crystal of the title compound, [ZnCl_2_(C_6_H_8_N_4_)_2_], consists of two Cl^-^ ions, two N atoms from two different pyrazines (N2, N6) and other two imine N atoms (N3, N7), forming a distorted octahedron (Fig. 1). The equatorial plane of the octahedron is occupied by Cl1, N2, N6 and Cl2, the four atoms are almost coplanar, with the dihedral angle of 13.28 (1)°. Atoms N3, N7 are in the axial positions of the octahedron. The distances between N3, N7 and Zn^II^ (2.158 (1) Å, 2.164 (1) Å) are shorter than those of N2, N6 of the equatorial plane with Zn^II^ (2.244 (1) Å, 2.258 (2) Å).

As shown in the packing diagram (Fig. 2), a three-dimensional framework is formed by intermolecular N—H···N and N—H···Cl interactions.

### Experimental

2-(1-hydrazinylideneethyl)pyrazine (Stadler *et al.*, 2010) (0.1 mmol, 0.0136 g) was dissolved in 20 ml absolute ethanol, then ZnCl_2_.2H_2_O (0.2 mmol, 0.0268 g) was added, and after 0.5 h of stirring at 333 K, the mixture was filtered and held at room temperature to allow slow evaporation of solvent. Shiny pale yellow crystals suitable for X-ray diffraction were collected after one week (Yield = 64%).

### Refinement

H atoms of the pyrazine ring were placed in calculated positions, with C—H = 0.93 Å, and refined using ariding modea, with *U*_iso_(H) = 1.2*U*_eq_(C). H atoms of the methyl group were located in difference Fourier maps and included as part of a rigid rotor, with C—H = 0.96 Å and *U*_iso_(H) = 1.5*U*_eq_(C methyl). H atoms of the NH_2_ groups were refined subject to a variable distance restraint that refined to N—H = 0.83 (2) Å and with *U*_iso_(H) = 1.5*U*_eq_(N).

### Crystal data

**Table:**

[ZnCl_2_(C_6_H_8_N_4_)_2_]	*F*(000) = 832
*M**_r_* = 408.60	*D*_x_ = 1.637 Mg m^−^^3^
Monoclinic, *P*2_1_/*n*	Mo *K*α radiation, λ = 0.71073 Å
Hall symbol: -P 2yn	Cell parameters from 4411 reflections
*a* = 8.4289 (8) Å	θ = 2.7–28.2°
*b* = 15.1128 (14) Å	µ = 1.81 mm^−^^1^
*c* = 13.4196 (13) Å	*T* = 296 K
β = 104.077 (1)°	Block, colourless
*V* = 1658.1 (3) Å^3^	0.20 × 0.18 × 0.15 mm
*Z* = 4	

### Data collection

**Table:**

Bruker APEXII CCD diffractometer	4132 independent reflections
Radiation source: fine-focus sealed tube	3456 reflections with *I* > 2σ(*I*)
Graphite monochromator	*R*_int_ = 0.026
φ and ω scans	θ_max_ = 28.3°, θ_min_ = 2.1°
Absorption correction: multi-scan (*SADABS*; Bruker, 2008)	*h* = −10→11
*T*_min_ = 0.713, *T*_max_ = 0.773	*k* = −18→20
11576 measured reflections	*l* = −9→17

### Refinement

**Table:**

Refinement on *F*^2^	Primary atom site location: structure-invariant direct methods
Least-squares matrix: full	Secondary atom site location: difference Fourier map
*R*[*F*^2^ > 2σ(*F*^2^)] = 0.029	Hydrogen site location: inferred from neighbouring sites
*wR*(*F*^2^) = 0.078	H atoms treated by a mixture of independent and constrained refinement
*S* = 1.03	*w* = 1/[σ^2^(*F*_o_^2^) + (0.042*P*)^2^ + 0.3057*P*] where *P* = (*F*_o_^2^ + 2*F*_c_^2^)/3
4132 reflections	(Δ/σ)_max_ = 0.009
223 parameters	Δρ_max_ = 0.42 e Å^−^^3^
4 restraints	Δρ_min_ = −0.21 e Å^−^^3^

### Special details

**Table:**

Geometry. All e.s.d.'s (except the e.s.d. in the dihedral angle between two l.s. planes) are estimated using the full covariance matrix. The cell e.s.d.'s are taken into account individually in the estimation of e.s.d.'s in distances, angles and torsion angles; correlations between e.s.d.'s in cell parameters are only used when they are defined by crystal symmetry. An approximate (isotropic) treatment of cell e.s.d.'s is used for estimating e.s.d.'s involving l.s. planes.
Refinement. Refinement of *F*^2^ against ALL reflections. The weighted *R*-factor *wR* and goodness of fit *S* are based on *F*^2^, conventional *R*-factors *R* are based on *F*, with *F* set to zero for negative *F*^2^. The threshold expression of *F*^2^ > σ(*F*^2^) is used only for calculating *R*-factors(gt) *etc*. and is not relevant to the choice of reflections for refinement. *R*-factors based on *F*^2^ are statistically about twice as large as those based on *F*, and *R*- factors based on ALL data will be even larger.

### Fractional atomic coordinates and isotropic or equivalent isotropic displacement parameters (Å^2^)

**Table:**

	*x*	*y*	*z*	*U*_iso_*/*U*_eq_	
Zn1	0.79801 (2)	0.074400 (13)	0.764996 (15)	0.03098 (8)	
Cl1	0.74457 (6)	0.17192 (3)	0.89354 (4)	0.04074 (12)	
Cl2	0.61902 (6)	−0.03984 (4)	0.78802 (4)	0.04526 (13)	
N1	1.2922 (2)	0.25410 (13)	0.75292 (16)	0.0535 (5)	
N2	1.00732 (17)	0.16076 (10)	0.75046 (11)	0.0322 (3)	
N3	1.01610 (18)	0.01092 (10)	0.85368 (11)	0.0331 (3)	
N4	1.0037 (3)	−0.06823 (12)	0.89868 (16)	0.0478 (5)	
H1N4	1.076 (3)	−0.0850 (16)	0.9461 (18)	0.057*	
H2N4	0.909 (3)	−0.0860 (17)	0.892 (2)	0.057*	
N5	0.8091 (3)	−0.09261 (13)	0.44379 (15)	0.0533 (5)	
N6	0.82991 (18)	−0.00119 (10)	0.62587 (11)	0.0327 (3)	
N7	0.65102 (18)	0.14165 (10)	0.63190 (12)	0.0330 (3)	
N8	0.5788 (2)	0.22084 (12)	0.64252 (16)	0.0473 (4)	
H1N8	0.493 (3)	0.2340 (17)	0.5969 (17)	0.057*	
H2N8	0.568 (3)	0.2228 (17)	0.7042 (15)	0.057*	
C1	1.1478 (3)	0.28105 (15)	0.69904 (19)	0.0508 (5)	
H1	1.1414	0.3328	0.6607	0.061*	
C2	1.0057 (2)	0.23502 (12)	0.69773 (16)	0.0405 (4)	
H2	0.9065	0.2567	0.6589	0.049*	
C3	1.1533 (2)	0.13197 (12)	0.80650 (14)	0.0329 (4)	
C4	1.2941 (2)	0.18004 (14)	0.80619 (18)	0.0459 (5)	
H4	1.3939	0.1593	0.8451	0.055*	
C5	1.1551 (2)	0.05001 (13)	0.86525 (14)	0.0333 (4)	
C6	1.3119 (2)	0.01689 (16)	0.93253 (16)	0.0478 (5)	
H6A	1.2922	−0.0376	0.9643	0.072*	
H6B	1.3546	0.0600	0.9847	0.072*	
H6C	1.3894	0.0070	0.8919	0.072*	
C7	0.9003 (3)	−0.12098 (14)	0.53299 (17)	0.0476 (5)	
H7	0.9594	−0.1731	0.5344	0.057*	
C8	0.9103 (3)	−0.07594 (12)	0.62350 (17)	0.0405 (4)	
H8	0.9752	−0.0986	0.6843	0.049*	
C9	0.7363 (2)	0.02954 (12)	0.53650 (14)	0.0321 (4)	
C10	0.7270 (3)	−0.01761 (14)	0.44637 (15)	0.0433 (5)	
H10	0.6608	0.0040	0.3854	0.052*	
C11	0.6464 (2)	0.11247 (12)	0.54143 (14)	0.0332 (4)	
C12	0.5611 (3)	0.15970 (16)	0.44551 (17)	0.0542 (6)	
H12A	0.4486	0.1689	0.4459	0.081*	
H12B	0.5671	0.1247	0.3868	0.081*	
H12C	0.6128	0.2159	0.4422	0.081*	

### Atomic displacement parameters (Å^2^)

**Table:**

	*U*^11^	*U*^22^	*U*^33^	*U*^12^	*U*^13^	*U*^23^
Zn1	0.02815 (12)	0.03323 (12)	0.02997 (12)	−0.00097 (8)	0.00402 (8)	0.00062 (8)
Cl1	0.0421 (2)	0.0393 (3)	0.0413 (3)	0.00117 (19)	0.01106 (19)	−0.00545 (19)
Cl2	0.0444 (3)	0.0473 (3)	0.0443 (3)	−0.0159 (2)	0.0113 (2)	−0.0026 (2)
N1	0.0427 (10)	0.0487 (11)	0.0738 (14)	−0.0098 (8)	0.0232 (10)	−0.0013 (9)
N2	0.0295 (7)	0.0318 (7)	0.0353 (8)	−0.0001 (6)	0.0078 (6)	−0.0032 (6)
N3	0.0339 (8)	0.0326 (8)	0.0309 (8)	0.0006 (6)	0.0044 (6)	0.0015 (6)
N4	0.0466 (11)	0.0423 (10)	0.0485 (11)	−0.0012 (8)	−0.0003 (9)	0.0142 (8)
N5	0.0660 (13)	0.0522 (11)	0.0441 (11)	0.0055 (9)	0.0181 (9)	−0.0093 (8)
N6	0.0328 (8)	0.0319 (8)	0.0326 (8)	0.0006 (6)	0.0064 (6)	0.0008 (6)
N7	0.0286 (7)	0.0313 (7)	0.0390 (9)	0.0020 (6)	0.0081 (6)	0.0001 (6)
N8	0.0458 (10)	0.0413 (10)	0.0545 (11)	0.0136 (8)	0.0115 (9)	0.0010 (8)
C1	0.0531 (13)	0.0376 (11)	0.0671 (15)	−0.0016 (9)	0.0250 (11)	0.0061 (10)
C2	0.0400 (10)	0.0351 (10)	0.0466 (11)	0.0033 (8)	0.0110 (8)	0.0030 (8)
C3	0.0275 (8)	0.0381 (10)	0.0334 (9)	−0.0009 (7)	0.0084 (7)	−0.0060 (7)
C4	0.0317 (10)	0.0515 (12)	0.0557 (13)	−0.0047 (9)	0.0130 (9)	−0.0018 (10)
C5	0.0290 (9)	0.0412 (10)	0.0284 (9)	0.0034 (7)	0.0047 (7)	−0.0043 (7)
C6	0.0331 (10)	0.0616 (14)	0.0448 (12)	0.0089 (9)	0.0018 (9)	0.0065 (10)
C7	0.0530 (13)	0.0399 (11)	0.0527 (13)	0.0083 (9)	0.0185 (10)	−0.0033 (9)
C8	0.0412 (11)	0.0369 (10)	0.0432 (11)	0.0056 (8)	0.0098 (9)	0.0018 (8)
C9	0.0300 (8)	0.0349 (9)	0.0319 (9)	−0.0030 (7)	0.0083 (7)	0.0019 (7)
C10	0.0498 (12)	0.0462 (11)	0.0330 (10)	0.0011 (9)	0.0079 (9)	−0.0016 (8)
C11	0.0293 (9)	0.0340 (9)	0.0343 (9)	−0.0004 (7)	0.0042 (7)	0.0054 (7)
C12	0.0656 (15)	0.0501 (13)	0.0408 (12)	0.0119 (11)	0.0011 (10)	0.0117 (9)

### Geometric parameters (Å, º)

**Table:**

Zn1—N3	2.1568 (15)	N8—H2N8	0.857 (18)
Zn1—N7	2.1637 (15)	C1—C2	1.382 (3)
Zn1—N2	2.2408 (15)	C1—H1	0.9300
Zn1—N6	2.2600 (15)	C2—H2	0.9300
Zn1—Cl2	2.3620 (5)	C3—C4	1.392 (3)
Zn1—Cl1	2.3934 (5)	C3—C5	1.466 (3)
N1—C1	1.320 (3)	C4—H4	0.9300
N1—C4	1.326 (3)	C5—C6	1.493 (3)
N2—C2	1.325 (2)	C6—H6A	0.9600
N2—C3	1.349 (2)	C6—H6B	0.9600
N3—C5	1.288 (2)	C6—H6C	0.9600
N3—N4	1.355 (2)	C7—C8	1.377 (3)
N4—H1N4	0.806 (19)	C7—H7	0.9300
N4—H2N4	0.827 (19)	C8—H8	0.9300
N5—C7	1.326 (3)	C9—C10	1.389 (3)
N5—C10	1.333 (3)	C9—C11	1.474 (3)
N6—C8	1.322 (2)	C10—H10	0.9300
N6—C9	1.348 (2)	C11—C12	1.494 (3)
N7—C11	1.283 (2)	C12—H12A	0.9600
N7—N8	1.366 (2)	C12—H12B	0.9600
N8—H1N8	0.850 (19)	C12—H12C	0.9600
			
N3—Zn1—N7	154.76 (6)	N2—C2—H2	119.2
N3—Zn1—N2	73.96 (6)	C1—C2—H2	119.2
N7—Zn1—N2	87.67 (5)	N2—C3—C4	119.51 (18)
N3—Zn1—N6	88.53 (6)	N2—C3—C5	117.46 (16)
N7—Zn1—N6	73.43 (6)	C4—C3—C5	123.03 (17)
N2—Zn1—N6	88.17 (5)	N1—C4—C3	122.96 (19)
N3—Zn1—Cl2	95.08 (4)	N1—C4—H4	118.5
N7—Zn1—Cl2	101.28 (4)	C3—C4—H4	118.5
N2—Zn1—Cl2	168.11 (4)	N3—C5—C3	115.63 (15)
N6—Zn1—Cl2	86.94 (4)	N3—C5—C6	124.36 (18)
N3—Zn1—Cl1	99.25 (4)	C3—C5—C6	120.01 (17)
N7—Zn1—Cl1	97.78 (4)	C5—C6—H6A	109.5
N2—Zn1—Cl1	89.67 (4)	C5—C6—H6B	109.5
N6—Zn1—Cl1	171.02 (4)	H6A—C6—H6B	109.5
Cl2—Zn1—Cl1	96.76 (2)	C5—C6—H6C	109.5
C1—N1—C4	116.34 (18)	H6A—C6—H6C	109.5
C2—N2—C3	117.43 (16)	H6B—C6—H6C	109.5
C2—N2—Zn1	129.32 (13)	N5—C7—C8	122.3 (2)
C3—N2—Zn1	113.23 (12)	N5—C7—H7	118.9
C5—N3—N4	121.17 (16)	C8—C7—H7	118.9
C5—N3—Zn1	119.41 (12)	N6—C8—C7	121.45 (19)
N4—N3—Zn1	119.40 (13)	N6—C8—H8	119.3
N3—N4—H1N4	120.3 (19)	C7—C8—H8	119.3
N3—N4—H2N4	114.6 (18)	N6—C9—C10	119.67 (18)
H1N4—N4—H2N4	120 (3)	N6—C9—C11	116.67 (16)
C7—N5—C10	116.23 (18)	C10—C9—C11	123.65 (17)
C8—N6—C9	117.76 (17)	N5—C10—C9	122.61 (19)
C8—N6—Zn1	128.07 (13)	N5—C10—H10	118.7
C9—N6—Zn1	113.55 (12)	C9—C10—H10	118.7
C11—N7—N8	119.22 (16)	N7—C11—C9	115.91 (15)
C11—N7—Zn1	119.93 (12)	N7—C11—C12	123.24 (18)
N8—N7—Zn1	120.38 (13)	C9—C11—C12	120.81 (18)
N7—N8—H1N8	117.0 (17)	C11—C12—H12A	109.5
N7—N8—H2N8	106.8 (17)	C11—C12—H12B	109.5
H1N8—N8—H2N8	114 (2)	H12A—C12—H12B	109.5
N1—C1—C2	122.2 (2)	C11—C12—H12C	109.5
N1—C1—H1	118.9	H12A—C12—H12C	109.5
C2—C1—H1	118.9	H12B—C12—H12C	109.5
N2—C2—C1	121.55 (19)		
			
N3—Zn1—N2—C2	178.63 (17)	C4—N1—C1—C2	0.0 (3)
N7—Zn1—N2—C2	16.15 (17)	C3—N2—C2—C1	0.6 (3)
N6—Zn1—N2—C2	89.63 (17)	Zn1—N2—C2—C1	178.73 (15)
Cl2—Zn1—N2—C2	155.39 (16)	N1—C1—C2—N2	−0.3 (3)
Cl1—Zn1—N2—C2	−81.65 (16)	C2—N2—C3—C4	−0.5 (3)
N3—Zn1—N2—C3	−3.15 (12)	Zn1—N2—C3—C4	−178.93 (14)
N7—Zn1—N2—C3	−165.63 (13)	C2—N2—C3—C5	179.72 (16)
N6—Zn1—N2—C3	−92.15 (13)	Zn1—N2—C3—C5	1.3 (2)
Cl2—Zn1—N2—C3	−26.4 (3)	C1—N1—C4—C3	0.1 (3)
Cl1—Zn1—N2—C3	96.57 (12)	N2—C3—C4—N1	0.1 (3)
N7—Zn1—N3—C5	50.1 (2)	C5—C3—C4—N1	179.9 (2)
N2—Zn1—N3—C5	5.20 (13)	N4—N3—C5—C3	175.73 (17)
N6—Zn1—N3—C5	93.72 (14)	Zn1—N3—C5—C3	−6.2 (2)
Cl2—Zn1—N3—C5	−179.49 (13)	N4—N3—C5—C6	−4.0 (3)
Cl1—Zn1—N3—C5	−81.77 (14)	Zn1—N3—C5—C6	174.13 (15)
N7—Zn1—N3—N4	−131.82 (16)	N2—C3—C5—N3	3.1 (3)
N2—Zn1—N3—N4	−176.68 (16)	C4—C3—C5—N3	−176.72 (18)
N6—Zn1—N3—N4	−88.16 (15)	N2—C3—C5—C6	−177.23 (17)
Cl2—Zn1—N3—N4	−1.36 (15)	C4—C3—C5—C6	3.0 (3)
Cl1—Zn1—N3—N4	96.35 (15)	C10—N5—C7—C8	0.0 (3)
N3—Zn1—N6—C8	23.01 (17)	C9—N6—C8—C7	0.4 (3)
N7—Zn1—N6—C8	−174.88 (17)	Zn1—N6—C8—C7	170.70 (15)
N2—Zn1—N6—C8	97.01 (17)	N5—C7—C8—N6	−0.5 (3)
Cl2—Zn1—N6—C8	−72.15 (16)	C8—N6—C9—C10	0.3 (3)
N3—Zn1—N6—C9	−166.32 (13)	Zn1—N6—C9—C10	−171.45 (14)
N7—Zn1—N6—C9	−4.21 (12)	C8—N6—C9—C11	179.32 (17)
N2—Zn1—N6—C9	−92.33 (13)	Zn1—N6—C9—C11	7.6 (2)
Cl2—Zn1—N6—C9	98.51 (12)	C7—N5—C10—C9	0.7 (3)
N3—Zn1—N7—C11	46.0 (2)	N6—C9—C10—N5	−0.8 (3)
N2—Zn1—N7—C11	88.76 (14)	C11—C9—C10—N5	−179.8 (2)
N6—Zn1—N7—C11	−0.03 (14)	N8—N7—C11—C9	176.15 (16)
Cl2—Zn1—N7—C11	−83.36 (14)	Zn1—N7—C11—C9	4.0 (2)
Cl1—Zn1—N7—C11	178.11 (14)	N8—N7—C11—C12	−1.8 (3)
N3—Zn1—N7—N8	−126.06 (16)	Zn1—N7—C11—C12	−174.00 (16)
N2—Zn1—N7—N8	−83.33 (14)	N6—C9—C11—N7	−7.9 (2)
N6—Zn1—N7—N8	−172.11 (15)	C10—C9—C11—N7	171.15 (18)
Cl2—Zn1—N7—N8	104.55 (14)	N6—C9—C11—C12	170.15 (18)
Cl1—Zn1—N7—N8	6.02 (14)	C10—C9—C11—C12	−10.8 (3)

### Hydrogen-bond geometry (Å, º)

**Table:**

*D*—H···*A*	*D*—H	H···*A*	*D*···*A*	*D*—H···*A*
N8—H2*N*8···Cl1	0.86 (2)	2.73 (2)	3.400 (2)	137 (2)
N8—H2*N*8···N1^i^	0.86 (2)	2.60 (2)	3.165 (3)	124 (2)
N4—H2*N*4···Cl2	0.83 (2)	2.60 (2)	3.250 (2)	137 (2)
N4—H1*N*4···Cl1^ii^	0.81 (2)	2.66 (2)	3.4429 (19)	165 (2)

Symmetry codes: (i) *x*−1, *y*, *z*; (ii) −*x*+2, −*y*, −*z*+2.

## References

[bb1] Adams, T. B., Doull, J. & Feron, V. J. (2002). *Food Chem. Toxicol.* **40**, 429–451. 10.1016/s0278-6915(01)00123-5 11893403

[bb2] Blackstock, A. W., Acostamadiedo, J. & Lesser, G. (2000). *Clin. Lung Cancer*, **2**, 62–66. 10.3816/clc.2000.n.019 14731342

[bb3] Bruker (2008). *SADABS*, *SAINT-Plus* and *APEX2* Bruker AXS Inc., Madison, Wisconsin, USA.

[bb4] Ha, T. G., Jang, J. J. & Kim, S. G. (1999). *Chem. Biol. Interact.* **121**, 209–222. 10.1016/s0009-2797(99)00094-0 10418965

[bb5] Lee, S. E., Chung, H. & Kim, Y. S. (2012). *Food Chem.* **131**, 1248–1254.

[bb6] Sheldrick, G. M. (2008). *Acta Cryst.* A**64**, 112–122. 10.1107/S0108767307043930 18156677

[bb7] Stadler, A. M., Puntoriero, F., Nastasi, F., Campagna, S. & Lehn, J. M. (2010). *Chem. Eur. J.* **16**, 5645–5660. 10.1002/chem.200900632 20379974

